# Retraction Note: Mechanistic insights into CDCA gene family-mediated glioblastoma progression: implications for diagnosis, prognosis, and therapeutic targeting

**DOI:** 10.1186/s41065-026-00749-9

**Published:** 2026-10-09

**Authors:** Chang Liu

**Affiliations:** School of Medicine, The Chinese University of Hong Kong, Shenzhen, Guangdong 518116 China

**Retraction Note: Hereditas 162, 43 (2025)**

https://doi.org/10.1186/s41065-025-00415-6

The Editor-in-Chief has retracted this article. After publication, concerns were raised regarding the in vitro images presented in Fig. 9 of this article, where the patterns of cells and colonies do not appear consistent with natural cell growth and proliferation. Additionally, the uncut western blot gel presented in the Supplementary Material appears to show contradictory results, with two proteins probed in adjacent lanes with different results. It has also been noted that a number of references in this article do not appear to support the statements preceding the citations.

The Editor-in-Chief therefore no longer has confidence in the presented data.

Chang Liu disagrees with this retraction.

